# Thermodynamic Efficiency of Somatic Exocytosis of Serotonin

**DOI:** 10.3389/fphys.2019.00473

**Published:** 2019-05-31

**Authors:** Paula Noguez, J. Miguel Rubí, Francisco F. De-Miguel

**Affiliations:** ^1^Instituto de Fisiología Celular-Neurociencias, Universidad Nacional Autónoma de México, Mexico City, Mexico; ^2^Facultat de Física, Universitat de Barcelona, Barcelona, Spain; ^3^Centro de Ciencias de la Complejidad, Universidad Nacional Autónoma de México, Mexico City, Mexico

**Keywords:** somatic exocytosis, extrasynaptic, serotonin, thermodynamic efficiency, vesicle transport, kinesin, myosin, leech

## Abstract

Through somatic exocytosis neurons liberate immense amounts of transmitter molecules that modulate the functioning of the nervous system. A stream of action potentials triggers an ATP-dependent transport of transmitter-containing vesicles to the plasma membrane, that ends with a large-scale exocytosis. It is commonly assumed that biological processes use metabolic energy with a high thermodynamic efficiency, meaning that most energy generates work with minor dissipation. However, the intricate ultrastructure underlying the pathway for the vesicle flow necessary for somatic exocytosis challenges this possibility. To study this problem here we first applied thermodynamic theory to quantify the efficiency of somatic exocytosis of the vital transmitter serotonin. Then we correlated the efficiency to the ultrastructure of the transport pathway of the vesicles. Exocytosis was evoked in cultured Retzius neurons of the leech by trains of 10 impulses delivered at 20 Hz. The kinetics of exocytosis was quantified from the gradual fluorescence increase of FM1-43 dye as it became incorporated into vesicles that underwent their exo-endocytosis cycle. By fitting a model of the vesicle transport carried by motor forces to the kinetics of exocytosis, we calculated the thermodynamic efficiency of the ATP expenses per vesicle, as the power of the transport divided by total energy ideally produced by the hydrolysis of ATP during the process. The efficiency was remarkably low (0.1–6.4%) and the values formed a W-shape distribution with the transport distances of the vesicles. Electron micrographs and fluorescent staining of the actin cortex indicated that the slopes of the W chart could be explained by the interaction of vesicles with the actin cortex and the calcium-releasing endoplasmic reticulum. We showed that the application of thermodynamic theory permitted to predict aspects of the intracellular structure. Our results suggest that the distribution of subcellular structures that are essential for somatic exocytosis abates the thermodynamic efficiency of the transport by hampering vesicle mobilization. It is remarkable that the modulation of the nervous system occurs at the expenses of an efficient use of metabolic energy.

## Introduction

Extrasynaptic exocytosis, namely the release of transmitters and peptides from the neuronal soma, dendrites, and axons, is a source of modulators of the activity of the nervous system (De-Miguel and Fuxe, 2012; De-Miguel and Nicholls, 2015). Most low molecular weight transmitters and different peptides are released extrasynaptically by exocytosis (for review see Trueta and De-Miguel, 2012). Molecules released in this manner diversify the responses of neural circuits to a given stimulus by integrating the activity of neurons, glia and capillaries (Fuxe et al., 2007; Rozanski et al., 2012; Hirasawa et al., 2015; Newman, 2015).

The mechanism for extrasynaptic exocytosis is substantially different from that for release at synapses and similar to the mechanism for exocytosis from endocrine cells. Somatic exocytosis involves a multi-regulated sequence of serial and parallel reactions that couple excitation with exocytosis (Ludwig and Stern, 2015; Del-Bel and De-Miguel, 2018). Vesicles -usually large and electrodense (see Trueta and De-Miguel, 2012) filled with signaling molecules, rest far from the plasma membrane. Bursts of electrical activity evoke a transmembrane calcium entry that triggers calcium release from the endoplasmic reticulum (review see Trueta and De-Miguel, 2012; Del-Bel and De-Miguel, 2018). The calcium wave induces an increase of cytosolic ATP that triggers the vesicle transport by the activation of molecular motors (Nicholls et al., 2003; Llorente-Folch et al., 2013; Rueda et al., 2014; Del-Pozo and De-Miguel, in preparation). Exocytosis starts as vesicles arrive at the plasma membrane (De-Miguel and Trueta, 2005). Calculations of the ATP expenses per vesicle fusing (ATP_ves_) made by applying thermodynamic theory to the kinetics of somatic exocytosis of serotonin, unveiled that the amount of ATP_ves_ has a W-shape dependence on the transport distance of the vesicles (De-Miguel et al., 2012). This relationship may reflect the cytoarchitecture of the transport pathway, since electron micrographs contain endoplasmic reticulum and mitochondria -which are engines for somatic exocytosis- often invading the transport pathway of the vesicles (Trueta et al., 2004, 2012; De-Miguel et al., 2012). In addition, the actin cortex at rest prevents the vesicle transport, but after the onset of electrical activity turns into a component of the transport system by binding to myosin motors (Wang and Hatton, 2006; Tobin and Ludwig, 2007). Such observations raised the possibility that somatic exocytosis operates under low thermodynamic efficiency, opposing to the common assumption that biological processes make a high efficiency use of their metabolic energy. Theoretically, when friction forces hamper the work, a fraction of the energy dissipates in the form of heat, reducing the thermodynamic efficiency of the process. For this reason, measurements of the amount of energy provide only a partial story; it is also necessary to estimate how efficiently the energy is used. This focal problem was studied here by making use of the advantages of the well-known somatic exocytosis of serotonin by the Retzius neuron of the leech.

**Figure 1:**
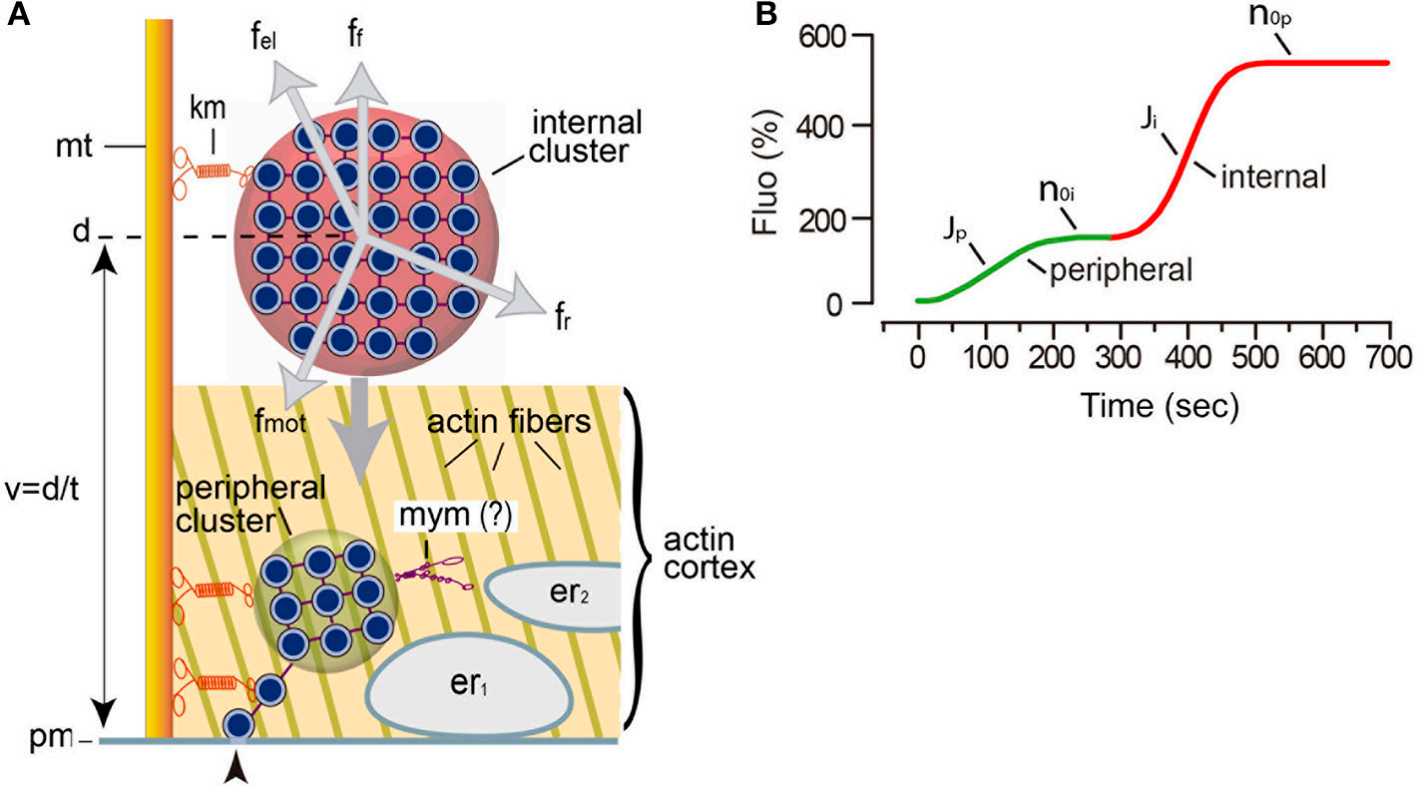
Mechanism for somatic exocytosis in the Retzius neuron. **(A)** Schematic representation of the vesicle transport system. The scheme incorporates findings from this study. Vesicle clusters rest at variable distances from the plasma membrane. More than one cluster can be attached to the same microtubule (mt) bundle by kinesin motors (km). Internal clusters (red) lay across the actin cortex. The transport of vesicle clusters depends on four forces: the motor forces (*f*_*mot*_), elastic forces (*f*_*el*_), friction forces (*f*_*f*_), and random forces (*f*_*r*_). At rest, these forces are in equilibrium. The motor forces are microtubule- and kinesin-dependent (De-Miguel et al., 2012). Electrical stimulation produces a cascade of calcium-dependent events that activate the motor forces. Vesicle clusters are transported to the plasma membrane along a distance (*d*), at a velocity (*v* = *d/t*). Peripheral clusters (green) rest immersed in the actin cortex at the time of stimulation, flanked by two layers of endoplasmic reticulum (er_1_, er_2_). Inside the actin cortex, myosin motors (mym) add to kinesin motors to transport the vesicle clusters to the plasma membrane (pm). The reduced number of vesicles as clusters approach the plasma membrane seems to obey a continuous vesicle detachment and transport to the plasma membrane (Leon-Pinzon et al., 2014). Fusion of these vesicles (arrowhead) produces the constitutive release of serotonin that permits the large-scale exocytosis when whole clusters arrive at the plasma membrane in response to electrical stimulation. **(B)** Biophysical parameters measured from the FM1-43 kinetics upon electrical stimulation of exocytosis. The green segment is the contribution of a peripheral cluster; the red segment is the contribution of an internal cluster. The latency to the onset of release contains information on the distance and velocity (*t* = *d/v*) of the vesicle transport. The *J* value is the flux of vesicles (vesicles fused per second). The *n*_*o*_ values are the number of vesicles that fused per vesicle cluster.

The large (~70 μm) soma of a Retzius neuron stores serotonin in astronomic numbers of 100 nm diameter electrodense vesicles (Coggeshall, 1972). At rest or following electrical stimulation with trains of 10 action potentials at 1 Hz (a frequency similar to the spontaneous firing of serotonergic neurons), vesicles remain clustered at variable distances from the plasma membrane and exocytosis does not occur (Figure 1; De-Miguel et al., 2012). By contrast, the same number of action potentials evoked at a high 20 Hz frequency triggers the massive transport of vesicle clusters to the plasma membrane and the large-scale exocytosis. Within the following hundreds of seconds, tens of thousands of vesicles distributed in ~90 clusters will discharge their content from different parts of the soma (Trueta et al., 2003; De-Miguel et al., 2012). The large-scale exocytosis is sustained for long periods in absence of further transmembrane calcium entry, by the serotonin that has been released (Leon-Pinzon et al., 2014). Activation of serotonin autoreceptors evoke an intracellular IP3-dependent release of calcium from the endoplasmic reticulum adjacent to the plasma membrane. This localized calcium elevation maintains exocytosis until the fusion of the last vesicles in the clusters (Leon-Pinzon et al., 2014). It is common that a second and larger cluster of vesicles arrives at the same release site producing a second bulk of exocytosis (De-Miguel et al., 2012; Leon-Pinzon et al., 2014). Figure 1 recapitulates the elementary aspects of this mechanism.

In this study we applied a thermodynamic theoretical approach to calculate the efficiency of somatic exocytosis of serotonin. As the reporter of exocytosis we used the fluorescence increase of FM1-43 dye, which stains the inside of vesicles that undergo their exocytosis/endocytosis cycle (Betz et al., 1992; De-Miguel et al., 2012). A mathematical approach applied to the kinetics of exocytosis permitted to calculate the transport distance, velocity and power of the whole process of somatic exocytosis from individual release sites. By incorporating new experimental data to the model fittings, we improved our previous estimates of the ATP expenses per vesicle fused (ATP_ves_). The thermodynamic efficiency of the ATP_ves_ expenses was calculated from the work in the presence of friction forces, divided by the ideal energy generated by the ATP cleavage. The thermodynamic efficiency along the traveling distance of the vesicles was correlated with the ultrastructure of the transport pathway, analyzed from electron micrographs, and fluorescent staining of the actin cortex.

## Materials and Methods

### Ethics Statement

Animal research was approved by the Animal Committee of the Instituto de Fisiología Celular, Universidad Nacional Autónoma de México.

### Isolation and Culture of Neurons

Experiments were made using Retzius neurons of the medicinal leech *Hirudo sp*. For experiments with FM1-43, neurons were individually isolated by suction through a glass pipette after an enzyme treatment (Dietzel et al., 1986) and plated in glass-bottom culture dishes coated with concanavalin-A (Sigma, St. Louis, MO). The culture medium was L-15 (Sigma) supplemented with 6 mg/mL glucose, 0.1 mg/mL gentamycin and 2% fetal bovine serum (Gibco, Gaithersburg, MD). Experiments were made at 18°C after 1–3 days in culture.

### Stimulation and Detection of Exocytosis

Exocytosis was quantified from the fluorescence increases produced by the incorporation of FM1-43 (2 μM; Molecular Probes) by the vesicles that undergo fusion and endocytosis (Betz and Bewick, 1993). Vesicle fusion permits the dye to enter the vesicles and stain their internal membrane. Endocytosis keeps the dye inside the vesicle. Therefore, each vesicle fusion contributes with a step to the fluorescence in the release site. We have previously shown that the large-scale exocytosis from vesicles arriving in a cluster lasts for hundreds of seconds, depending on the number of vesicles in the distance and the velocity of the transport. Upon endocytosis, the stained vesicles accumulate near the plasma membrane without being recycled to the releasable pool. Instead, they get packed forming multivesicular bodies that are later transported to the perikaryon (Trueta et al., 2012). Therefore, the gradual increase of fluorescence reflects accurately the kinetics of exocytosis; the plateau is reached once all the electrodense vesicles in the cluster have fused and the intensity of fluorescence indicates the number of vesicles that fused (De-Miguel et al., 2012). If any stained vesicle fuses again the amount of fluorescence does not increase because the vesicle is already stained.

Stimulation of exocytosis consisted of trains of 10 action potentials produced by intracellular injection of 10 ms current pulses at 20 Hz delivered every 2 min. The amplitude of the current pulses was adjusted in every neuron between 5 and 8 nA, to guarantee that each pulse in the train produced one action potential. The intracellular voltage was recorded using a Getting pre-amplifier operating in bridge balance mode. In eight other neurons the actin cortex was uncoupled by incubation with 1 μM cytochalasin D (Sigma-Aldrich) for 30 min at 18°C before stimulation.

Individual neurons were focused on their equator with a Nikon Eclipse TE 200 inverted microscope using a Nikon 100x oil immersion objective (NA 1.40). FM1-43 was excited at 488 nm and its fluorescent emission was measured at 543 nm. Images were taken every 2 s with a cooled CCD camera (IMAGO, Till Vision, Germany) at a 640 × 480-pixel resolution. Processing was made offline by using TILLvisION software.

The fluorescence measurements were made by linear interpolation, after non-specific fluorescence in each sequential image was subtracted from the intensity of the membrane. The area of release was estimated from the length of membrane in which fluorescence increased, by assuming that it was circular (De-Miguel et al., 2012). This number served as a reference for the model estimates of the number of vesicles (see Equation 1).

### Exclusion Criteria

Neurons commonly moved after electrical stimulation. Therefore, the focus of each spot in the time series analysis had to be confirmed from a z axis scanning of the neurons at the end of the experiment. Only fluorescent spots that remained in focus during the whole experiment or that could be aligned digitally were included in the analysis. Clusters extending for more than 1 μm of membrane length were excluded to avoid out-of-focus fluorescent emissions due to large numbers of vesicles (De-Miguel et al., 2012). For these reasons, we eliminated from our analysis a vast number of observations. However, the data from nine different neurons in which subsequent uncorrelated clusters of vesicles arrived at the same spot were a fair representation of the population.

### Quantitative Analysis of Exocytosis

**Figure 2:**
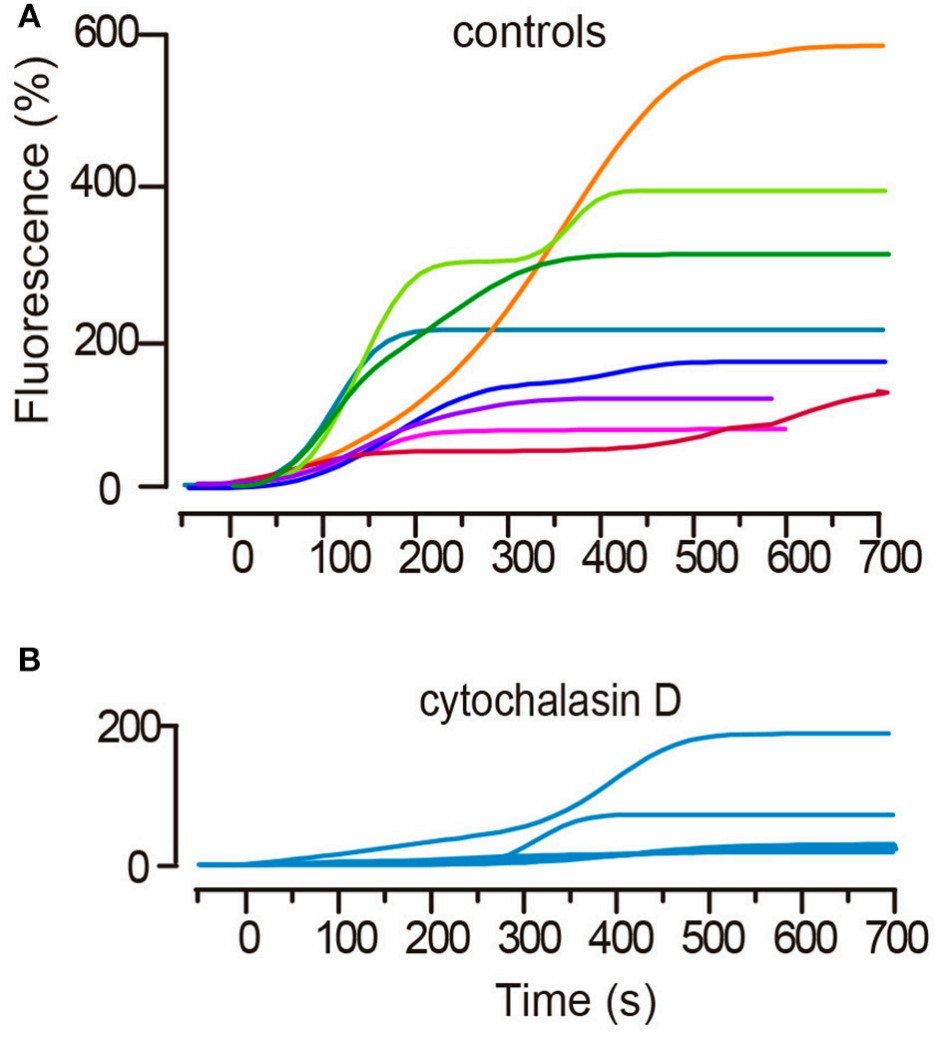
Kinetics of the large-scale exocytosis as measured from the fluorescence increases of FM1-43. **(A)** Superimposition of the FM fluorescence (Fluo) kinetics of eight representative spots from an equal number of neurons. Stimulation occurred at *t* = 0. A subsequent stimulation train was delivered every 2 min. Fluorescence is plotted as the percentage increase over the baseline. Subsequent sigmoidal increases appear in most kinetics, with each reflecting exocytosis from a subsequent vesicle cluster. **(B)** Treatment of neurons with cytochalasin D that uncouples the actin cortex delayed and reduced the fluorescence increases in response to stimulation and abolished the second sigmoidal fluorescence increase. The plot superimposes six kinetics from an equal number of neurons. Note the difference in the fluorescence scale bars between **(A,B)**.

The model was developed based on the kinetic properties of the FM1-43 fluorescence increases during exocytosis (Figure 2). A full account of the theoretical development of the model is presented in De-Miguel et al. (2012). In brief, the fluorescence kinetics *F*_(*t*)_ is proportional to the flux *J*_*ves*_ of vesicles arriving at the plasma membrane. The process of exocytosis is orders of magnitude faster than the speed of the vesicle transport. Therefore, the flux reflects the transport and can be defined as the number of vesicles fusing per second at an area *A* of the plasma membrane, according to:

(1) $$\begin{array}{r} F\left(t\right)=F_{0}+Ab\int_{0}^{t}J_{ves}\left(d,t\prime \right)dt\prime \end{array}$$

In Equation (1), *F*_0_ is the basal fluorescence produced by the interaction of the fluorescent dye with the extracellular layer of the plasma membrane; *b* is a proportionality factor relating the amount of fluorescence contributed by each vesicle. This proportionality stems from the fact that the internal membrane surface of the population of electrodense vesicles can be considered constant. The parameter “*t*” is the time, while “*d”* is the transport distance from the center of mass of the resting cluster to the plasma membrane. The distance was estimated by the model and was correlated with distances measured from electron micrographs of neurons stimulated with 1 Hz trains of impulses (De-Miguel et al., 2012). The plateau of the fluorescence increase indicates the total the number of vesicles (*n*_0_*)* that fused (Figure 1B). The parameters used in the model are in Table 1. The contribution of the distance, velocity and number of vesicles to the kinetics of fluorescence are presented schematically in Figure 1.

**Table 1:** Definition of the parameters used in the model.

**Symbol**	**Definition**	**Dimensions**
*A*	Area of a release site measured from the diameter of the fluorescence increase	L^2^
*ATP_*ves*_*	Number of ATP molecules hydrolyzed per vesicle fused.	_
β	Dimensionless constant referring to the fluorescence increase per vesicle fused	_
*d*	Transport distance of vesicle clusters measured from the center of mass of the cluster to the plasma membrane	L
*ΔG*	Gibb's free energy	ML^2^T^−2^
*ΔG_*ATP*_*	Gibb's free energy of ATP hydrolysis	ML^2^T^−2^
*F_0_*	Baseline value of the fluorescence intensity	a.u.
*F(t)*	Fluorescence as a function of time	a.u.
*F*	Force exerted by the motors	MLT^−2^
*f_*f*_*	Friction forces per mass unit	LT^−2^
*f_*r*(*t*)_*	Random forces due to thermal agitation per mass unit	LT^−2^
*f_*el*_*	Elastic forces per mass unit	LT^−2^
*f_*mot*_*	Molecular motor force per mass unit	LT^−2^
*Fv*	Power of the large-scale exocytosis	ML^2^T^−3^
*J*_*ves*_	Current density of vesicles	L^−2^T^−1^
κ	Elastic constant of the cytoskeleton	MT^−2^
*m*	Mass	M
η	Thermodynamic efficiency	_
*n_*o*_*	Number of vesicles per cluster	_
*r*	Rate constant of ATP hydrolysis	T^−1^
*v*	Average velocity of vesicle cluster transport	LT^−1^
ω	Characteristic frequency of the elastic force	T^−1^
*W*	Work of the large-scale exocytosis	ML^2^T^−2^

As shown also in Figure 1, the flux of vesicles, *J*_*ves*_, depends on four forces. The first two are a frictional force due to the resistance opposed by the medium to the vesicle motion and a random force produced by thermal agitation. These two forces produce the Brownian motion of the vesicles at the experimental temperature. The third force is elastic and confines the individual vesicles in a cluster. These forces are considered harmonic because the vesicle displacements they produce are small. This assumption simplifies the model. The fourth force is used by the motors to carry the vesicle clusters plus mitochondria at an average velocity “*v*_*ves*_” (Svoboda and Block, 1994; Schnitzer and Block, 1997). The Force of the motor and the elastic forces acting together on the vesicles can be defined as:

(2) $$\begin{array}{r} F=f_{el}+f_{mot}=\omega^{2}\left(d-vt\right) \end{array}$$

Where ω is the characteristic frequency at which the elastic forces confine the vesicles, as described by:

(3) $$\begin{array}{r} \omega =\sqrt{\frac{k}{m}} \end{array}$$

We consider that the vesicle motion toward the cell membrane is unidimensional and can be described in terms of the distance coordinate. Therefore, *J*_*ves*_ depends on the friction and motor forces plus the contribution of diffusion, where *J*_*ves*_ = ρ*v*_*ves*_ and $v_{ves}=\beta^{-1}F$. Then *J*_*ves*_ can also be expressed as:

(4) $$\begin{array}{r} J_{ves}=-D\frac{\partial \rho }{\partial x}+\beta^{-1}\rho F \end{array}$$

where the term $-D\frac{\partial \rho }{\partial x}$ accounts for diffusion, with D the being the diffusion coefficient, and the second term is the product of the friction (β^−1^ρ) and the motor (*F*) forces, due to the effect of the driving force (De-Miguel et al., 2012).

These forces are modeled by a linear function of the position, which corresponds to a harmonic force. Harmonic forces yield in general an oscillatory motion in which the velocity of the particle decreases until it vanishes at a certain point, and then increases in the opposite direction. In this case the harmonic force acts only during forward motion. Therefore, that sole force can model the main movement of the vesicles to the plasma membrane.

### Improved Estimates of ATP Consumption

The work that moves the vesicle cluster-mitochondria complex along a certain distance equals the change in free energy:

(5) $$\begin{array}{r} W_{tot}=k\left(d^{2}-dvt\right)=\Delta G_{process} \end{array}$$

In our previous calculations, the elastic (*k*) constant value of the molecular motors was a free parameter in the model. For such reason there were variations in its predicted value from one release site to another (De-Miguel et al., 2012). In this study, we fed the model with a unique *k* = 0.027 pN/nm value measured experimentally by Bruno et al. (2011).

Equation (6) expresses the total amount of energy provided by the cleavage of ATP that is necessary under ideal conditions, to transport a vesicle cluster for a certain distance without dissipation. Due to the heterogeneity of the vesicle cluster sizes, the energy is expressed as the number of ATP molecules hydrolyzed per vesicle fused (*ATP*_*ves*_) by dividing the numerator by the number of vesicles in the cluster *n*_0_:

(6) $$\begin{array}{r} ATP_{ves}=\frac{\Delta G_{process}}{n_{0}\Delta G_{ATP}} \end{array}$$

The Δ*G*_*ATP*_ value was 5.4 × 10^−20^ Joules (Alberty and Goldberg, 1992), and *n*_0_ is the number of vesicles that fused (see Equation 1). Table 2 compares the data obtained for the elastic and motor forces, and for the *ATP*_*ves*_ expenses reported here and in our previous study (De-Miguel et al., 2012). Table 2 shows that the *ATP*_*ves*_ values presented here remained within similar orders of magnitude than those previously reported.

**Table 2:** Ranges of parameter values contributing to the vesicle transport.

**Cluster**	**Peripheral**	**Internal**
*η_0_*	98–490	52–930
*d* (μm)	0.6–2.1	1.9–6.2
*v* (nm/s)	15.0–95.0	11.5–60.0
*J*_*ves*_ (ves/sec)	0.2–4.0	0.9–10.33
Elastic force (N × 10^−11^)	1.2–4.2	3.8–12.0
	(0.6–4.1)	(2.2–24.2)
Motor force (N × 10^−10^)	1.2–6.0	1.8–4.7
	(0.6–4.4)	(1.4–3.5)
*ATP_*ves*_*	10.9–49.4	13.6–260
	(3.4–73.0)	(0.4–25.1)
Efficiency (%)	0.2–6.4	0.1–1.9

*Data in red are the estimates calculated in this study; data in parenthesis are from our previous study (De-Miguel et al., 2012)*.

### Thermodynamic Efficiency

Since energy dissipation due to the presence of frictional forces is expected to occur in a non-ideal transport by living neurons, we used the classical definition of efficiency as the ratio between the ideal and the lost work and applied as in De Groot and Mazur (1969), later applied to analyze the efficiency of the calcium ATP_ase_ by Lervik et al. (2012).

(7) $$\begin{array}{r} \eta =\frac{W_{ideal}-W_{lost}}{W_{ideal}} \end{array}$$

The *W*_*ideal*_ is the given by:

(8) $$\begin{array}{r} W_{ideal}=-r\Delta_{r}G \end{array}$$

Where *r* is the force-related rate of ATP cleaved by the molecular motors. The kinetics of force generation of molecular motors are consistent with a two-step reaction (Higuchi et al., 1997). The binding of ATP has a rate constant that depends on the concentration and therefore its units are μM^−1^·s^−1^; a second rate constant *r* accounts for the force generation used here, and has s^−1^ units (Higuchi et al., 1997). The *r* value related to the force generation is the one that matters for our estimates, since according to De Groot and Mazur (1969, Equation 10 of Chapter X), it relates the reaction rate to the production of entropy. This definition is adequate to calculate the efficiency since the entropy production related to thermal dissipation. As in our previous equation, Δ_*r*_*G* is the Gibbs free energy of the ATP cleavage.

The work lost (*W*_*lost*_) can be obtained from the Gouy-Stodola theorem in Equation (7) (see Kjelstrup et al., 2010):

(9) $$\begin{array}{r} W_{lost}=\;T\sigma \end{array}$$

where *T* is the constant temperature of the surroundings of the system and σ is the production of entropy that accounts for the thermal dissipation. In our case, the energy is dissipated during the transport of vesicles and in the process of the ATP hydrolysis at a rate given by:

(10) $$\begin{array}{r} T\sigma =-r\Delta G-J_{ves}\Delta \mu_{ves} \end{array}$$

This expression was derived according to energy conservation along the transport path; Δμ_*ves*_ in a previous paper was an electroneutral exchange of ions by the calcium ATP_ase_ (22), and here it relates to the force F of the motors. We also considered a negligible thermal driving force (Lervik et al., 2012). This expression was also used by Caplan and Essig (1999), who considered the isothermal case and the linear regime of proper pathways.

The efficiency η of vesicle exocytosis can be given in terms of measurable quantities as:

(11) $$\begin{array}{r} \eta \;=\frac{-r\Delta G-T\sigma }{-r\Delta G}\;=\;\frac{J_{ves}\Delta \mu_{ves}}{-r\Delta G} \end{array}$$

The numerator of this fraction involves the flux of particles *J*_*ves*_ and the chemical potential difference Δμ_*ves*_ between the initial and the final position of the vesicles, which corresponds to a force. Since in the experiments we follow the trajectories of the vesicles, the numerator that corresponds to the work applied to the vesicles to move with a velocity *v*_*ves*_ is given by the product *F*_*ves*_*v*_*ves*_. The efficiency is then:

(12) $$\begin{array}{r} \eta \;=\frac{F_{ves}v_{ves}}{r|\Delta G|} \end{array}$$

where *F*_*ves*_ is the sum of elastic and motor forces given in Equation (2).

The efficiency per vesicle was calculated by dividing by the number of vesicles (*n*_0_) in the cluster.

(13) $$\begin{array}{r} \eta =\frac{F_{ves}v_{ves}\;}{n_{0}r{|\Delta G}_{ATP}|} \end{array}$$

The rate constant *r* of the force generated by the ATP hydrolysis for kinesin motors is < 100–300 s^−1^ (Higuchi et al., 1997; Cross, 2004). A similar range of values has been measured for myosin motors (Johnson and Taylor, 1978; De La Cruz et al., 1999). Therefore, the 100 s^−1^ value used here may account for either motor type acting separately and with non-additive effects.

### Curve Fitting and Statistical Analysis

The kinetics of exocytosis of 19 release sites obtained from six neurons were fitted to the mathematical model, as in De-Miguel et al. (2012; see **Figure 5**). The traveling distance and number of vesicles were limited by the ranges of values determined previously from electron microscopy (De-Miguel et al., 2012). In this way we reduced the possible combinations of values estimated by the model and reduced the error in the fittings. The data were fitted manually by using our own routines made in MATLAB software (MathWorks, Massachusetts, USA). This procedure was preferred over minimum square methods because sigmoidal functions with two crossovers became smoothed in abrupt parts of curves that had strong contributions by the motor activity. A previous account on the quality of the fittings and the effects of varying the values of the free parameters has been presented in De-Miguel et al. (2012).

The data sets relating the ATP_ves_ expenses and the efficiency vs. distance presented in **Figure 6** were fitted to empirical polynomial functions to underscore the W-shape of the charts. The order of the polynomials was adjusted to obtain the best *R*^2^ correlation values. The polynomial functions in our previous study had better fittings; data of peripheral clusters were fitted by a third-order polynomial function with *R*^2^ = 0.92. Likewise, the fitting quality of the internal cluster data was *R*^2^ = 0.83. The reason for the lower quality of the fittings presented here is the absence of data in the distance ranges of the minimum peaks (see **Figure 6A**). However, the polynomial fittings had no other intention than to give numeric support to the clear W-shaped trend of the data. In any case, due to the calculation improvements already described, we consider that the data sets in this study are more precise.

### Staining of the Actin Cortex

The actin cortex was measured from fluorescent stainings made by use of phalloidin coupled to a fluorescent probe. Neurons were fixed right after stimulation with CytoSkelFix (Cytoskeleton) for 4 min at −20°C and permeabilized for 30 min with phosphate-buffer saline (PBS) solution containing 0.3% Triton X-100. SFB (10%) was added as a blocker for 2 days, during which the preparations were maintained at 4°C. Actin was stained by incubation for 30 min at 37°C with phalloidin coupled to Alexa-fluor 488 (0.16 μM, Invitrogen). Controls incubated in parallel without phalloidin were devoid of any staining.

Observations of fluorescence made using an Olympus Fluoview FV1000 upright confocal scanning microscope equipped with a 60X oil immersion objective (1.45 NA). Images were stored digitally using Fluoview 3.1 software (Olympus). The thickness of the actin cortex was measured from digital images using ImageJ software (NIH, USA).

### Electron Microscopy

Neurons in isolated ganglia were fixed right after stimulation with 10 trains of 10 impulses at 1 or 20 Hz, delivered at 10 s intervals. The procedure to obtain thin (70–100 nm) sections for electron micrographs of Retzius cells were as in Kuffler et al. (1987). Sections were photographed in a JEOL 1010 electron microscope (JEOL USA Inc.).

## Results

### Kinetics of Somatic Exocytosis

Electrical stimulation of Retzius neurons with 20 Hz trains produced FM1-43 fluorescence spots at the neuronal surface (Trueta et al., 2003). In this experimental situation each release site develops a fluorescent spot upon the subsequent fusion of electrodense vesicles. A first sigmoidal fluorescence increase (Figure 2A) reflects the fusion of vesicles forming a cluster that rested at distances <2.0 μm from the plasma membrane (De-Miguel et al., 2012). Such clusters are named “peripheral” and data are presented as green symbols (Figure 2B). A second sigmoidal increase in the same spot is due to fusion of vesicles in clusters that traveled from more internal sites (see De-Miguel et al., 2012). These clusters are called “internal,” and data are represented with red symbols (Figure 2B).

The model fittings predicted that 52–930 vesicles per cluster became fused in the 19 clusters analyzed from 10 neurons. There was no correlation between the number of vesicles, the distance, or the velocity of pairs of clusters arriving subsequently at the same release site (*R*^2^ ≤ 0.4 for all cases). For example, in a release site the peripheral cluster contained 95 vesicles that traveled along 0.6 μm at 70 nm/s, whereas the internal cluster contained 262 vesicles that traveled along 4.1 μm at 12 nm/s; in other release site the peripheral cluster also had 95 vesicles that traveled along 0.6 μm at 59 nm/s, but the internal cluster contained 675 vesicles that traveled along 2.7 μm at 18 nm/s. In a third release site the peripheral cluster was larger than the internal, however both traveled at the same 30 nm/s velocity. Data of the pairs of clusters can be seen in **Figure 10**.

It was previously shown that uncoupling microtubules by addition of colchicine to the neurons abolishes somatic exocytosis, most likely by uncoupling the actin-mediated transport (De-Miguel et al., 2012). In addition, the actin cortex in neurons and secretory cells imposes a barrier for the transport at rest, but electrical activity in the presence of myosin turns the actin cortex to an active transporter (Vitale et al., 1995; Lang et al., 2000; Oheim and Stühmer, 2000; Giner et al., 2005; Tobin and Ludwig, 2007; Gutiérrez and Gil, 2011; Torregrosa-Hetland et al., 2011). For its potential friction-generating qualities, we uncoupled the actin cortex to test its contribution to the ATP expenses and efficiency of the transport. Pre-incubation of six neurons with cytochalasin D in the bathing solution produced that in all but one case (*n* = 8 clusters from 6 neurons), the latency to the onset of exocytosis was increased from 50–80 s to ~300 s. In addition, the amplitude of the plateau fluorescence was reduced to values corresponding to 40–107 vesicles fused (Figure 2B). It can also be seen that in all but one case the fluorescence increase lacked the second sigmoidal. This experiment shows that the actin cortex has a sophisticated contribution to somatic exocytosis.

### The Rate of Vesicle Fusion Reflects the Transport Conditions

**Figure 3:**
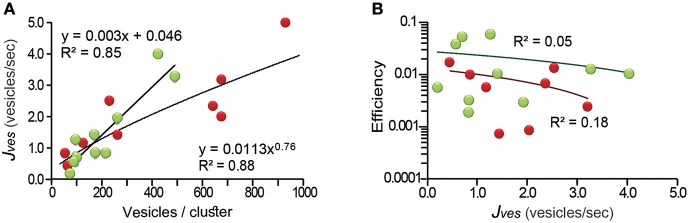
The thermodynamic efficiency of the ATP per vesicle fused was independent from the rate of vesicle fusion (*J*_*ves*_). **(A)** The *J*_*ves*_ values related linearly to the number of vesicles in peripheral clusters (green), and with a 0.76 exponent to the number of vesicles of internal clusters (red; for explanation see text). **(B)** The efficiency did not correlate with the rate of fusion of internal or external clusters (black lines), respectively. The equations giving the best fit to the data and the significance levels are presented.

The calculations of the efficiency depended on the correct measure of the flux of vesicles (*J*_*ves*_; see Equations 1 and 4 in the Methods section), indirectly representing the rate of exocytosis. For this reason, it was necessary to demonstrate that the area of membrane did not limit exocytosis. This possibility was confirmed by correlating *J*_*ves*_ with the number of vesicles that fused. The *J*_*ves*_ values ranged from 0.2 to 5.2 vesicles per second in the 19 clusters studied and had a linear relationship (*R*^2^ = 0.85) with the area of release (Figure 3A). For internal clusters the relation was sublinear with a 0.76 exponent (*R*^2^ = 0.88). The deviation from linearity occurred when the number of vesicles per cluster was above 600. Such large clusters may have encountered major constrains in their traveling path, as will be confirmed below. However, the large clusters can extend over wide areas of membrane, as seen in electron micrographs (Trueta et al., 2004). Therefore, we consider that the *J*_*ves*_ values reflect accurately the transport velocity of the vesicle clusters. In support to this, Figure 3B shows that the efficiency of the ATP expenses was uncorrelated with the *J*_*ves*_ values.

### ATP-Dependence of the Thermodynamic Efficiency

**Figure 4:**
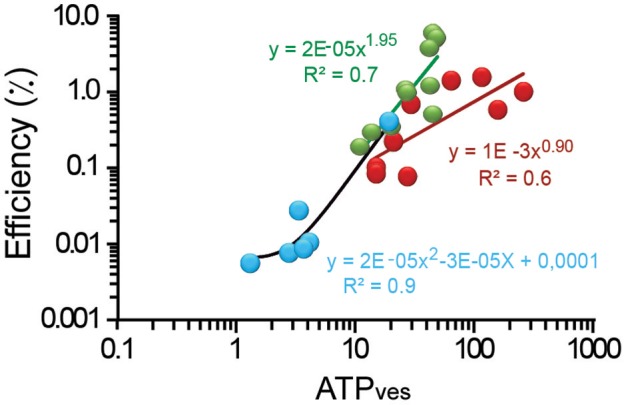
Efficiency as a function of the ATP_ves_ expenses. Logarithmic plot of the efficiency as a function of the ATP_ves_. The thermodynamic efficiency of exocytosis from peripheral clusters had a quadratic relationship with ATP_ves_ expenses (green line); internal clusters (red) the relationship had a 0.9 exponent (dark red line). Uncoupling the actin cortex with cytochalasin D (blue dots) reduced the range of the relationship by two logarithmic units. Data was fitted by a quadratic potency function (black line). The equations and their significance values are presented.

The efficiency of the ATP_ves_ expenses of peripheral clusters spanned within more than one logarithmic unit from values as low as 0.2% for the smallest 11 ATP_ves_ value. From there, the efficiency increased to its largest 6.4% value when the ATP_ves_ cost was 49 molecules (Figure 4). The intermediate data were described by a supralinear relationship. The 1.9 potency slope (*R*^2^ = 0.7) is consistent with the possible incorporation of a second type of motor, presumably myosin coupled to the actin cortex. This addition could increase the efficiency of ATP_ves_ expenses by incorporating the characteristic longer steps per ATP molecule of myosin motors (Kohler and Rohrbach, 2015). The internal vesicle clusters were distributed within an efficiency range from 0.1 to 1.9% and a large range of ATP_ves_ cost of 14 to 260. The plot approximated a linear relationship (*R*^2^ = 0.6) with a 0.9 exponent. This result is more consistent with the action of a single type of motor (kinesin) mediating the transport (Kohler and Rohrbach, 2015).

Uncoupling the actin cortex with cytochalasin D reduced the range of ATP_ves_ expenses to 1.3–19, and the efficiency to the lowest 0.06–0.4% range in the study (Figure 4). The small amount of exocytosis in this experimental situation suggests that only vesicles inside the cortex at the time of stimulation could fuse. This is in agreement with the 0.37–2.7 μm transport distances estimated for these clusters. The potency function that fitted these data, had again a slope of two (*R*^2^ = 0.90). These data in addition with the short transport distances points to the possibility that short filaments of actin bound to myosin and contributed to move the proximal clusters that fused. Our conclusion from these sections is that the efficiency values are proportional to the ATP_ves_ expenses and may indicate additive effects on the vesicle transport.

### Velocity as a Reporter of the Thermodynamic Efficiency

**Figure 5:**
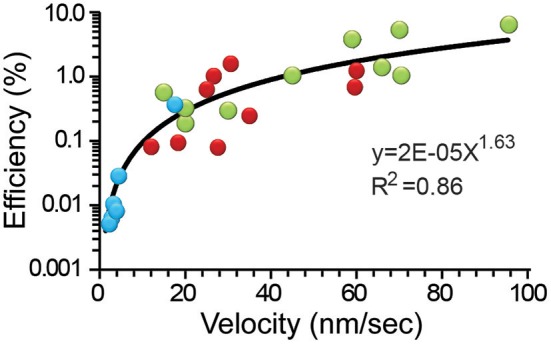
The efficiency depended on the transport velocity. Data from internal (red), peripheral (green) clusters, and after addition of cytochalasin D (blue) were pooled together and fitted to a potency function (black line). The equation and significance level are presented.

Figure 5 shows that the thermodynamic efficiency displayed a power law relationship with the transport velocity of the vesicles. This trend incorporated the whole population of data, although peripheral clusters traveled with a wider 15–95 nm/s range of velocities. The velocities above 60 nm/s were unreachable for internal clusters. As expected from Equation (12), the peripheral vesicles transported at higher velocities also displayed the largest -although yet low- thermodynamic efficiencies between 4.2 and 6.4%. This finding is not surprising from a different perspective: the peripheral clusters were small and had less mass to oppose to frictive obstacles. At velocities below 60 nm/s there was an extensive overlapping of both cluster populations in the chart, with the efficiencies of the internal clusters being ≤1.9%. Application of the actin depolymerizing agent cytochalasin D produced a reduction in the transport velocity to 1.5–17.0 nm/s that correlated with the lower 0.006–0.04% range of efficiency. This result seems more related to the lack of one motor system than to an increase in the frictive forces. Data from the internal and external clusters could be fitted independently to potency functions with equal 1.56 exponents and *R*^2^ values of 0.69 and 0.41, respectively. However, the pooled data set, including those data collected in the presence of cytochalasin D were best described by a single function with a 1.63 slope (*R*^2^ = 0.86). This property anticipates that the distribution of velocities expresses effects from other variables such as the size of the vesicle cluster and the cytoarchitecture of the pathway. To complement this scheme, we have also shown that the ATP_ves_ cost also increases with the velocity (De-Miguel et al., 2012).

### Distance-Dependence of the ATP Expenses

**Figure 6:**
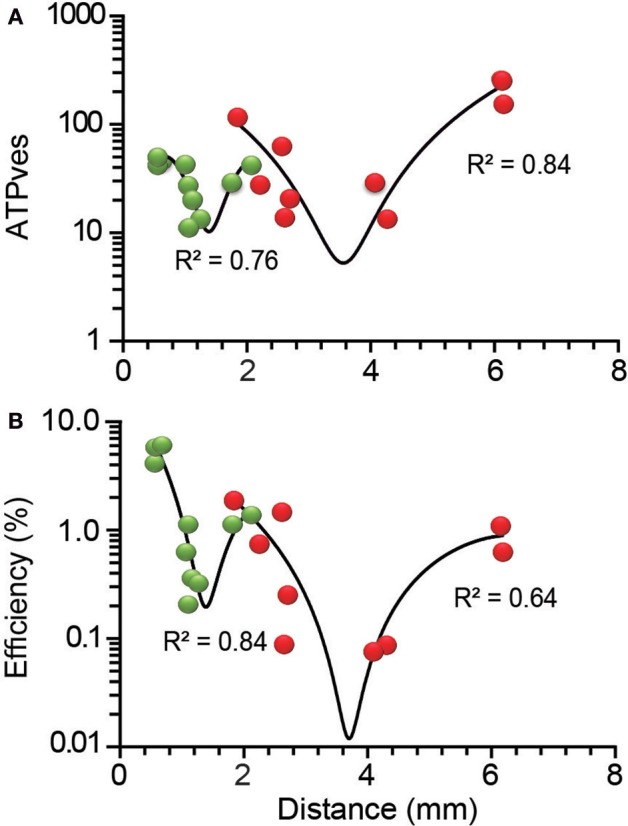
Distance-dependence of the ATP_ves_ expenses and thermodynamic efficiency of exocytosis. **(A)** The ATP_ves_-dependence on the distance had a W-shape. The short distance range of the plot contains information from peripheral (green) clusters, while distances above 1.9 μm contain information from internal (red) clusters. A maximum intermediate peak forming a high energy barrier appeared by 1.9 μm, with convergence of peripheral and internal clusters. **(B)** The distance-dependence of the thermodynamic efficiency of somatic exocytosis also was W-shaped, with each vesicle population separated by the intermediate maximum. The clusters that rested at the shortest distances had the largest efficiency values. The *R*^2^ values are the significance of the polynomial functions fitted to the data (black lines).

The distance-dependence of the ATP_ves_ expenses reproduced the previously described W-shape (De-Miguel et al., 2012), with certain differences in the ranges of ATP_ves_ values, that are presented in Table 2. The transport of external clusters along the shortest 0.6 μm resting distances had a high 52 ATP_ves_ cost. As the transport distance increased to 1.3 μm, the ATP_ves_ cost became gradually reduced to reach minimum of 13 ATP_ves_. As the distance continued to increase, the trend of the energy cost reversed to reach 44 ATP_ves_ when the distance was 2.1 μm. This was the distance at which the high-energy barrier of the W curve appeared. This was also the site of convergence for external and internal clusters. The first internal cluster rested at 1.9 μm and release from its vesicles occurred at a high 118 ATP_ves_ cost (Figure 6A). From there, the ATP_ves_ expenses had a second decrease to reach the second minimum, which in this case was 14 ATP_ves_ in the segment between 2.3 and 4.3 μm. The last increase complemented the W shape by reaching the largest 260 ATP_ves_ value at 6.15 μm (Figure 6A). The polynomial fittings in the plots had the unique intention to underscore the W-shape distribution of the data without any use for the model calculations.

### Distance-Dependence of the Thermodynamic Efficiency

As expected from the proportionality of the efficiency and the ATP_ves_ values in Figure 4, the thermodynamic efficiency also displayed a W-shape dependence on the transport distance (Figure 6B). The distance ranges of the efficiency plots were the same as for the ATP expenses. However, the efficiency of peripheral clusters covered a larger range of values than that of internal clusters. The first polynomial distribution in Figure 6B included the data from peripheral clusters (*R*^2^ = 0.84). The low distance extreme had the higher 6.4% efficiency. At deeper distances the values first decreased to a 1.9% minimum and then increased to reach the intermediate 1.4% peak right where peripheral and internal clusters intersected. The second part of the chart had a sole contribution from internal clusters. This section could be approached by a polynomial function (*R*^2^ = 0.64). The 0.08% second minimum efficiency occurred between 2.65 and 4.30 μm, although as discussed above, data with even lower values may be missing. At 6.15 μm the efficiency reached its internal maximum value of 1.1%. Our hypothesis to explain the W-distribution of the ATP_ves_ expenses and efficiency was that each component of the plot reflects a mechanical or ultrastructural interference on the vesicle pathway. An increase in the friction can be produced by a larger number of vesicles being transported or by obstacles in the pathway. It is worth to remark that the slopes of the W chart do not refer to the behavior of the vesicles within the range of distances covering that slope but indicate the amount of ATP expenses and efficiency over their whole trip to the membrane. Following this idea, those vesicles traveling the longest distances would have to overcome the obstacles that produce each of the four slopes of the chart. These possibilities were explored in the following parts of the study.

### Ultrastructural Correlates of the Thermodynamic Efficiency

**Figure 7:**
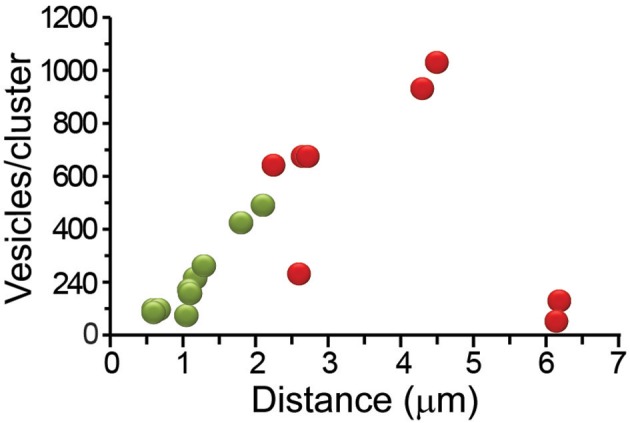
Distance-dependence of the number of vesicles that fused per cluster. The number of vesicles in the clusters increased with the distance from the plasma membrane. The most internal clusters were small. Peripheral clusters are represented in green and internal in red.

For its susceptibility to be affected by friction forces, the number of vesicles in the clusters was our first candidate to explain the components of the W chart. The plot in Figure 6B showed that the vesicle clusters near the plasma membrane had the largest efficiencies. Figure 7 shows that those same clusters were small, as explained in Figure 1. Intuitively, the transport of small cargos occurs with higher efficiency owing to the smaller counteracting friction forces. However, the ATP_ves_ cost of the transport of these clusters was among the highest (Figure 6A). Moreover, the relationship between the size of the clusters and the efficiency did not follow the W shape. The number of vesicles per cluster increased as the clusters rested more internally, to reach 930 vesicles at 4.5 μm from the plasma membrane. Then the last cluster carried only 62 vesicles with a 1.1% efficiency. This small cluster was most likely being assembled near a Golgi apparatus (see Trueta et al., 2012). This result points to elements other than the vesicle numbers affecting the efficiency. Based on this reasoning we carried out a search for ultrastructural elements affecting the thermodynamic efficiency.

### Ultrastructure of the Actin Cortex

**Figure 8:**
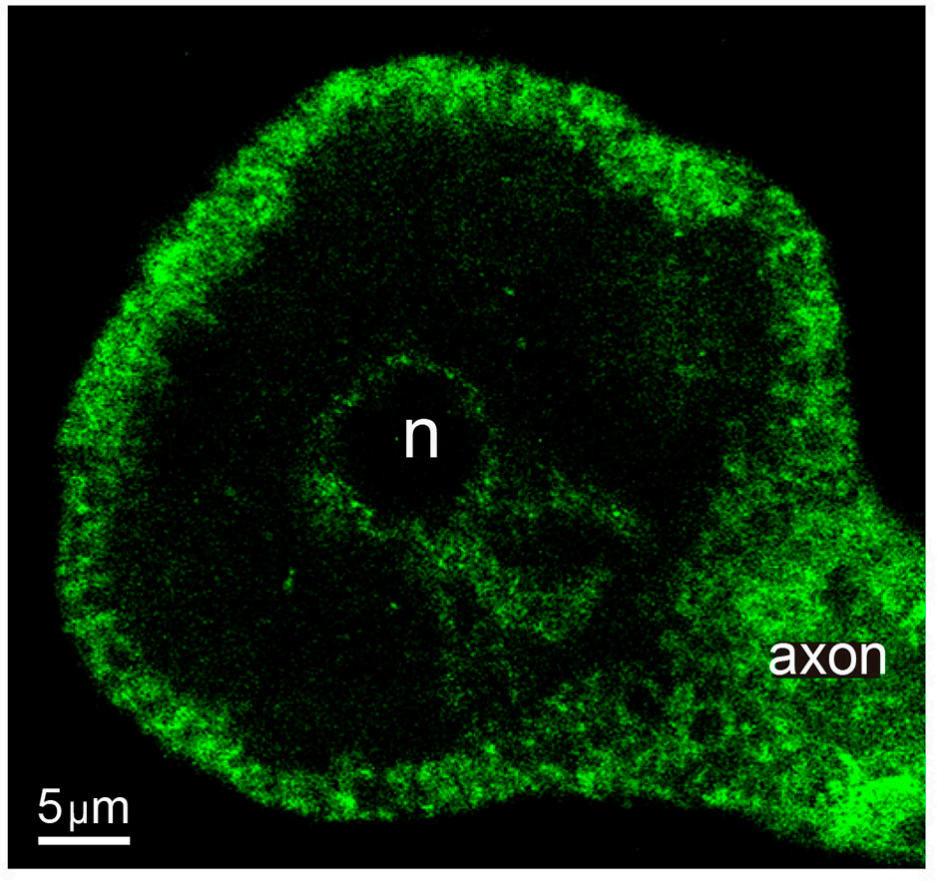
Actin cortex of Retzius neurons. Confocal equatorial image of the soma of a Retzius neuron showing the fluorescence of Alexa dye coupled to phalloidin. The perinuclear region and primary axon also display a profuse staining. n indicates the position of the nucleus.

The dual role of the actin cortex on the vesicle transport already described suggested its influence on the thermodynamic efficiency of the transport. The actin cortex of neurons stimulated with 1 Hz trains of impulses was stained with phalloidin coupled to Alexa fluorescent dye. The fluorescent label in equatorial images of the soma consisted of a thick 1.93 ± 0.74 μm (S.D.) array of radial bundles (Figure 8), as quantified from 46 counts of different parts of the cortex of five neurons. This thickness was similar to the distance at which we found the high energy barrier and high efficiency intermediate peak of the W distributions shown in Figure 6. Such value gives a possible explanation to the third slope of the W distributions in Figure 6. As internal clusters rested closer to the high energy barrier increasing amounts of vesicles were inside the actin cortex, where the myosin carried by the clusters assembles the actin-myosin transport with the actin filaments (Noguez et al., in preparation). As larger fraction of the clusters added the myosin-actin dymers to the kinesin-tubulin transport, the cost and efficiency of the transport would increase. However, this is a partial explanation. A fuller account comes from the origins of the two short distance slopes of the W charts, which are explained in the following paragraphs.

### Distribution of Endoplasmic Reticulum

**Figure 9:**
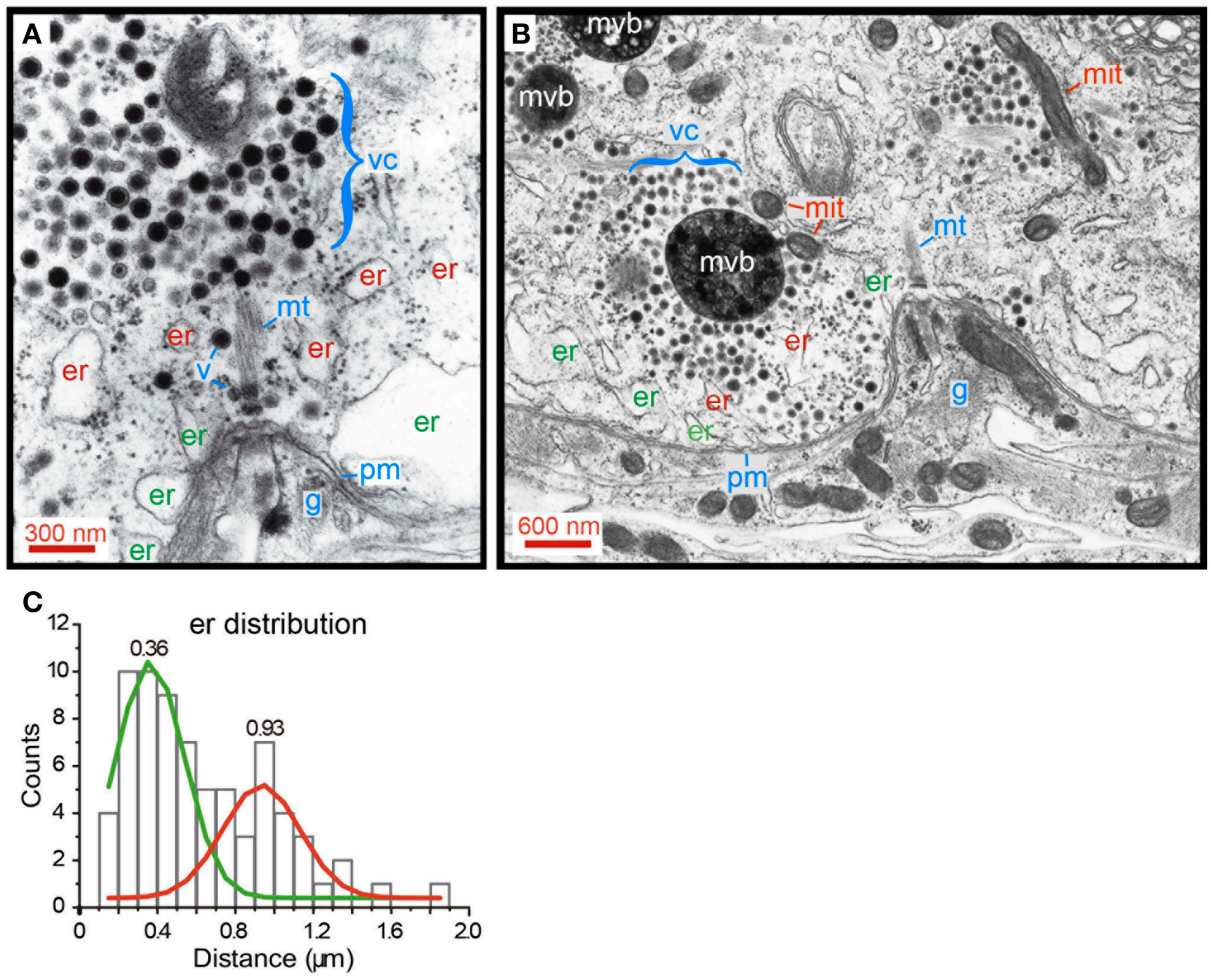
Distribution of smooth endoplasmic reticulum in the soma shell. **(A)** Electron micrograph of a neuron that had been stimulated at 1 Hz. Vesicles remain at their resting states. A somatic vesicle cluster (vc) is bound to the membrane (pm) via a bundle of cytoskeletal fibers, most likely microtubules (mt). Peripheral endoplasmic bags (er) rest closely apposed to the plasma membrane. More internally, another layer of endoplasmic reticulum (er) contains smaller bags. Individual vesicles (v) can be seen in an apparent migration to the membrane, along the microtubule bundles (see also Figure 1). Glial cell processes (g) in an invagination of the Retzius neuron, right at the anchor site of microtubules to the plasma membrane. **(B)** Electron micrograph of a release site in a neuron that had been stimulated with 20 Hz trains. Vesicles in a large cluster are apposed to the membrane. Transport occurs through a bottleneck of endoplasmic reticulum. Mitochondria (mit) are common satellites of the clusters that travel with them. Multi-vesicular bodies (mvb) result from the incorporation of vesicles after exo/endocytosis (Trueta et al., 2012). Note that exocytosis occurs onto the glia. **(C)** Distribution of endoplasmic reticulum in the soma shell. The distances have the plasma membrane as reference. Peripheral (er) and internal (er) pools of endoplasmic reticulum were distinguished by Gaussian fittings to the histograms. The mean values for each distribution are indicated.

The search for alternative ultrastructural candidates to explain the ATP_ves_ expenses and thermodynamic efficiency of the first half of the W distributions took us to the endoplasmic reticulum. Electron micrographs showing release sites of neurons were obtained by fixing neurons right after 1 Hz stimulation (*n* = 5) to maintain the resting ultrastructure of the transport pathway (Trueta et al., 2004). The images in Figure 9 contain structures immersed in the actin cortex, although the conventional fixation technique that we used to prepare the sections does not allow to resolve its structure. Figure 9A contains a cluster of electrodense vesicles resting distantly from the plasma membrane. Subsequent vesicles are bound by small fine filaments. The vesicle cluster was bound indirectly to the plasma membrane via a thick bundle of microtubules, according to the diameter of the individual fibers. Single vesicles appeared along the microtubules, presumably on their way to the plasma membrane (Leon-Pinzon et al., 2014; Figure 1). As explained in Figure 1, this vesicle migration explains the reduction of the number of vesicles as the peripheral clusters rest closer to the plasma membrane. An alignment of endoplasmic reticulum bags bordered the intracellular side of the plasma membrane; deeper and smaller bags of endoplasmic reticulum were scattered between the vesicles and the plasma membrane. One may wonder that vesicles being transported to the plasma membrane will find these bags on their way forming a bottleneck.

Electron micrographs obtained from neurons fixed after 20 Hz stimulation confirmed this idea. The image in Figure 9B shows vesicles from a large cluster reaching the plasma membrane by passing between endoplasmic reticulum. These obstacles bottlenecked the flux of vesicles and can be considered as sources of friction forces that reduce the thermodynamic efficiency of somatic exocytosis.

Countings of the distance between the plasma membrane and the center of mass of endoplasmic reticulum bags are presented in Figure 9C. The green and red lines fit the data distribution to two Gaussians, one with a mean 0.36 ± 0.11 μm (SD) distance from the plasma membrane, corresponding to the more external layer of endoplasmic reticulum. The red line fits the distribution of the scattered internal bags of endoplasmic reticulum, rendering a mean 0.93 ± 0.12 μm distance. This distribution supports that the endoplasmic reticulum is a source of friction forces that reduce the thermodynamic efficiency. Smaller vesicle clusters facing the plasma membrane seem to rest between the external layer of endoplasmic reticulum in absence of further obstacles to reach the plasma membrane.

### Multiple Variables Affect the Thermodynamic Efficiency

**Figure 10:**
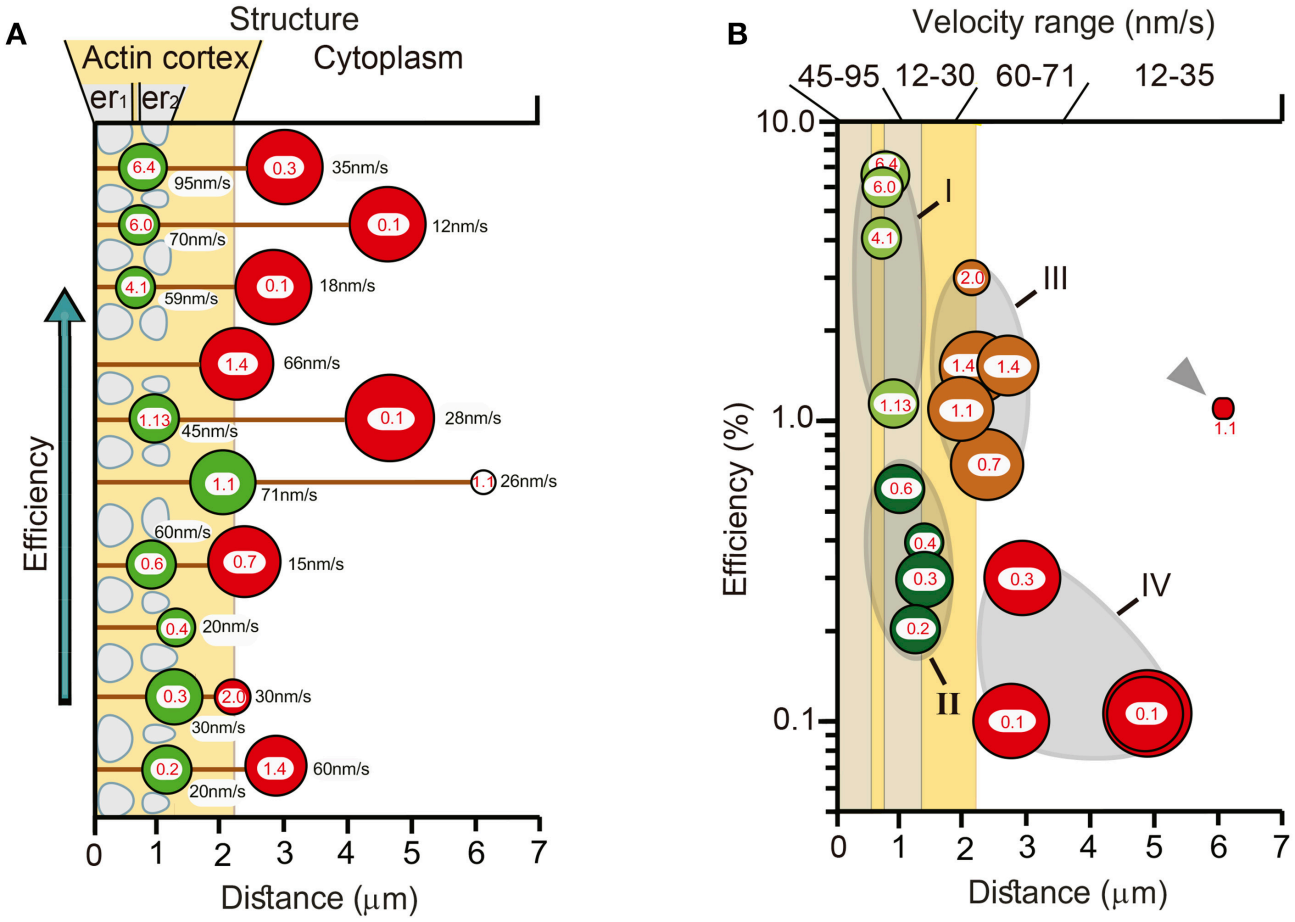
Structural and biophysical correlations of the thermodynamic efficiency of the ATP_ves_ hydrolysis. **(A)** Pairs formed by peripheral (green circles) and internal (red circles) vesicle clusters that discharged in the same site are represented attached to microtubules (black lines). The distances were calculated by the model fittings from the mass center of the cluster to the plasma membrane. The diameter of the clusters corresponds to the number of vesicles in the cluster. For each cluster, the red numbers inside indicate the efficiency (%), and the black numbers on the right the transport velocity (nm/sec). The peripheral clusters are presented from bottom to top according to their increasingly efficiency values. Their paired internal clusters had independent efficiency values, velocities, sizes and traveling distances. The actin cortex is represented in pale orange. Both layers of endoplasmic reticulum, one immediately inside the plasma membrane (er_1_) and the other ~1 μm inside (er_2_) are represented as pale sacs. The number of vesicles decreased as the clusters approached the plasma membrane. The efficiency of the peripheral clusters decreased as the distance increased. The number of vesicles, the efficiency, the transport distance and velocity of their corresponding internal clusters were uncorrelated with those of their peripheral pairs. **(B)** Vesicle clusters were groped according to their efficiency, size, distance, and velocity. The thermodynamic efficiency of somatic exocytosis also correlated with the thickness of the actin cortex (pale orange) and both subsequent layers of endoplasmic reticulum (gray bars). Group I contains the smallest and more peripheral clusters, which also had the highest thermodynamic efficiency values. These clusters rested at the entrance of the submembrane barrier of endoplasmic reticulum (gray bar adjacent to the plasma membrane). Group II contained clusters embedded within the second layer of endoplasmic reticulum, (more internal gray bar). The transport velocities and efficiencies of these clusters were smaller than those of Group I. Group III (brown) rested in the limits of the actin cortex. These clusters were large, moved with high velocity and their efficiencies were above 1%. Group IV (red) contained large internal clusters that rested inside the actin cortex. Their velocities were smaller than those in Group III. The smaller cluster was the deeper (gray arrowhead). This cluster did not correlate with the rest of group IV for its size and for having a relatively large 1.1 efficiency, in spite of its low 26 nm/s velocity. Its size and position suggest that it was in the assembly process at the time of the stimulation series.

The multivariable schemes in Figure 10 integrate the data on the possible variables that determine the thermodynamic efficiency of somatic exocytosis. Each element of the transport pathway has been scaled based on the experimental evidence. The diameter of the circles representing each cluster was calculated from its number of vesicles. Peripheral clusters are organized in an ascending order of efficiency. The distance from the plasma membrane to the center of mass of the clusters was determined by the model fittings. Pairs of clusters releasing in the same spot are associated by a black line representing the microtubule bundles. The efficiency of the ATP expenses per vesicle is indicated in the center of each cluster; the velocity of the transport is indicated on the right of each cluster. The actin cortex and endoplasmic reticulum are represented according to the morphometric analysis descried above. At first glance there is a good correlation between the size, the resting distance and the efficiency of peripheral clusters. The thermodynamic efficiency of the exocytosis process from vesicles in these peripheral clusters was inversely proportional to their resting distance. Similar correlations are unclear for the internal clusters in this representation, because although they were associated to a peripheral cluster, their biophysical properties were not correlated. The correlations between different variables of the clusters and the ultrastructure of the transport pathway are simplified in Figure 10B. The vesicle clusters were grouped from I to IV based on the distance, efficiency, size, velocity, and ultrastructural interactions. Both regions in which the ATP hydrolysis and efficiency had increasing slopes also had higher transport velocities. On the contrary, the regions in which the energy and efficiency had decreasing trends had slower velocity ranges. This result, which can be predicted from the formula of the thermodynamic efficiency in Equation (12), acquires a morphological correlate in Figure 10. Table 3 summarizes the data according to the groups of vesicle clusters. For the small peripheral clusters in Group 1, the additive action of two motors and the clusters resting already between endoplasmic reticulum in the more peripheral layer may favor higher velocities and efficiencies at the expenses of the high ATP_ves_ cost. Group II has two friction barriers represented by the two layers of endoplasmic reticulum. As vesicles enter the barriers the thermodynamic efficiency decreases. This can be attributed to the friction exerted by the endoplasmic reticulum on the vesicle clusters. In group III, the large clusters are at least in part, immersed in the actin cortex. The assembly of two motors may increase the ATP expenses and efficiency. However, the effect of the endoplasmic reticulum barriers is diminished. This can be explained if the flow of the peripheral clusters that precede the larger internal clusters has already diminished the friction forces by displacing the endoplasmic reticulum to the side, thus widening the spaces though which the internal clusters will flow. If so, the pairing of clusters in the same microtubule pathway would favor the transport of the internal larger vesicle clusters. The ATP expenses and thermodynamic efficiency of vesicles in Group IV may stem from their longer traveling distance, part of which is solely covered by the force of kinesin motors. In addition, entering the actin cortex may impose friction forces due to the accommodation of the vesicles within the entanglement of the actin fibers, before the coupling of the myosin motors. Another group that can be predicted contains the small clusters that are assembled and transported from the parikaryon (see Trueta et al., 2012). However, more evidence is required to strengthen this argument.

**Table 3:** Variables contributing to the energy cost and efficiency of somatic exocytosis.

**Cluster group**	**I**	**II**	**III**	**IV**
Distance to membrane (μm)	0.6–1.1	1.0–2.3	1.8–2.6	2.7–6.1
Efficiency (0%)	1.0–6.4	0.2–0.6	0.7–2.0	1.1
ATP_ves_	27–45	11–43	28–260	13–30
Velocity nm/s	45–95	12–30	60–71	35–12
Size of cluster	Small	Intermediate	Large	Larger
Vesicles per cluster	95–169	174–260	229–641 (65)	262–930 (52)
Intracellular structures	Actin cortex, Endoplasmic reticulum 1	Actin cortex, Endoplasmic reticulum 1+2	Actin cortex interface Endoplasmic reticulum 1+2	Cytoplasm Actin cortex interface Endoplasmic reticulum 1+2
Transport system	Kinesin Myosin	Kinesin Myosin	Kinesin Some Myosin	Kinesin Later Myosin

*The red numbers correspond to the decreasing slopes of the W distributions of ATP_ves_ expenses and efficiency; the blue numbers correspond to the increasing slopes of the W distribution. Other colors underscore different structures; the tones indicate gradations. The number of vesicles in parenthesis are out of range. The value in Group IV corresponds to the most internal cluster, most likely under formation at the time of the stimulation of exocytosis. Endoplasmic reticulum 1 and 2 correspond to the external and internal layers in Figures 9, 10, respectively*.

## Discussion

The application of thermodynamic theory to the study of somatic exocytosis predicts how the cytoarchitecture of the vesicle trafficking pathway influences the cost and thermodynamic efficiency of serotonin release. In spite of the common belief that biological systems operate with high efficiency levels, somatic exocytosis is far from an ideal system. Isolated myosin motors moving on actin fibers have efficiencies of 45% (Kjelstrup et al., 2013). Therefore, the low efficiency reported here shows the additive effects of the low efficiency of the motors and the frictive effects imposed by the ultrastructure of the transport pathway. Surprisingly, the thermodynamic efficiency of the simple calcium ATPase immersed in the plasma membrane, depends on the lipid composition of the membrane, with low efficiency values ≤12% (Lervik et al., 2012). Therefore, the values estimated here seem reasonable by considering the number of variables affecting the vesicle transport.

It is pertinent to discuss a potential source of error in our measurements. The reports of the rate constant values of the ATP cleavage that fuels the actin and myosin motors have considerable variations depending on the subtype of motor and the experimental conditions of the measurements. Since these variations span over similar ranges 100–300 s^−1^, we could choose one single value to represent both rate constants for our calculations. The choice for the low 100 s^−1^ value seemed the most accurate for our purposes, because the 18°C temperature at which our experiments were carried out—leeches are cold blooded animals—decreases the rate constants of chemical processes. We have no way to justify that this choice reflects the real values with which the vesicle transport operates in Retzius neurons since their motor subtypes are only superficially known. Antibodies against myosin type IV succeeded to bind myosin in other set of experiments and the contribution of each motor to somatic exocytosis has been blocked pharmacologically (Noguez et al., in preparation), but we still lack estimates of their rate constants. If the rate constant values were higher, the estimate of the efficiency would decrease linearly in proportion to the increase in the rate constant values, as predicted from Equation (12). For example, a 20% increase in the rate constant value from 100 to 125 s^−1^ would reduce the efficiency from 6.0 to 4.8%; likewise, a 200% increase in the rate constant value to 300 s^−1^ -which is near the largest estimate available (Johnson and Taylor, 1978; Apell et al., 1996; Higuchi et al., 1997; De La Cruz et al., 1999; Cross, 2004; De la Cruz and Ostap, 2009)- would reduce the efficiency to 0.2%. In any case, the values would move downwards linearly but not logarithmically. The additional possibility that each motor operates with a different rate constant value cannot be resolved with the tools of our study. The measures of the average velocity of the transport normalize these possible variations without sensitivity to separate them.

A duality has emerged between the structure/function relationship of the release sites and the thermodynamic efficiency of somatic exocytosis. Each slope of the W distribution of the efficiency vs. distance plot is influenced by the interaction of vesicles with a particular building block of the exocytosis process. The same structures that regulate the exocytosis machinery hamper the thermodynamic efficiency of the transport. For example, the endoplasmic reticulum is the fundamental source for intracellular calcium that first activates the vesicle transport in response to high frequency stimulation (Trueta et al., 2004), and later maintains the large-scale exocytosis in response to the activation of serotonin autoreceptors (Leon-Pinzon et al., 2014). In a previous study (Trueta et al., 2004) we have shown that very large vesicle clusters become apposed to the plasma membrane in response to electrical stimulation. This observation implies that they occupy positions that at rest contained endoplasmic reticulum. The endoplasmic reticulum may then be moved aside during transport of the peripheral vesicle cluster. As a positive consequence, the internal cluster that follows the same trajectory may find a cleared pathway to the plasma membrane, with a concomitant saving of energy and reduced heat dissipation.

The actin cortex adds another duality to neurons and excitable endocrine cells by preventing the entry of the vesicle clusters at rest and by propelling vesicles in response to electrical stimulation (Vitale et al., 1995; Lang et al., 2000; Oheim and Stühmer, 2000; Giner et al., 2005; Tobin and Ludwig, 2007; Gutiérrez and Gil, 2011; Torregrosa-Hetland et al., 2011). The third player is mitochondria, the fuel generator that in response to calcium produces ATP that activates the motors. Mitochondria travel along with the vesicle clusters, as suspected because both organelles appear together in electron micrographs after 1 or 20 Hz stimulation. The cargo imposed by the mitochondria may be as large as, or even larger than that imposed by the cluster of vesicles. Therefore, it is most likely that the mobilization of the energy generators along with the vesicle clusters increases the friction forces at the expenses of the thermodynamic efficiency.

A final question from our study concerns its general significance. Somatic exocytosis is part of a wider communication complex system that involves not only the soma, but every part of the neuron. A remarkably similar mechanism for somato-dendritic exocytosis has been demonstrated in thalamic neurons releasing the peptides vasopressin and oxytocin (Ludwig and Stern, 2015). Moreover, the mobilization of serotonin-containing exocytosis structures in the isolated soma of Raphe neurons from mammals follow similar steps (Sarkar et al., 2012). The soma and dendrites of thalamic neurons releasing peptides contain an actin cortex whose activation promotes the vesicle transport and exocytosis (Tobin and Ludwig, 2007). The ATP expenses and efficiency of exocytosis have not been explored in those or in other types of neurons, but the highly conserved mechanism of somatic and in general extrasynaptic exocytosis (Trueta et al., 2012) suggest that the effects presented here may be of general interest. The dendrites and small axon varicosities, for example release of serotonin through a mechanism similar to that in the soma, although the transport distances of the vesicles are downscaled. The ATP expenses and the thermodynamic efficiency of the vesicle transport for their exocytosis may be scaled accordingly. Such smaller traveling distances may require less ATP and may operate with higher thermodynamic efficiencies.

The mechanism for somatic exocytosis seems to be highly conserved also for most signaling molecules in the central and peripheral nervous system (For review see Trueta and De-Miguel, 2012; Del-Bel and De-Miguel, 2018). Secretory cells follow similar mechanistic rules by transporting vesicles across an actin cortex in response to stimulation (Vitale et al., 1995; Lang et al., 2000; Oheim and Stühmer, 2000; Giner et al., 2005; Gutiérrez and Gil, 2011; Torregrosa-Hetland et al., 2011). These arguments suggest that our results represent general mechanisms in biology.

## Author Contributions

PN and FD-M designed and carried out the experiments, conducted the analysis and prepared the figures. MR designed the mathematical approach. FD-M wrote the manuscript.

### Conflict of Interest Statement

The authors declare that the research was conducted in the absence of any commercial or financial relationships that could be construed as a potential conflict of interest.
